# From guesswork to guidance: a delphi study on practices of talent identification and development in Para athletics

**DOI:** 10.3389/fspor.2026.1791259

**Published:** 2026-05-11

**Authors:** Annmarie Carroll, Alison O’Riordan, Kate Pumpa

**Affiliations:** 1 School of Public Health, Physiotherapy, and Sport Science, University College Dublin, Dublin, Ireland; 2 Paralympics Ireland, Irish Sport HQ, Dublin, Ireland; 3 Institute for Sport and Health, University College Dublin, Dublin, Ireland; 4 Queen Mary University of London, London, United Kingdom

**Keywords:** expert (Delphi) survey, Para athletes, Para athletics, Para sport, talent identification & development

## Abstract

**Objectives:**

To determine best practices in Para athletics talent identification and development through a Delphi survey.

**Methods:**

A three round Delphi survey was conducted with experts in Para athletics talent identification and development. Round One aimed to elicit insights into the current practices and beliefs in talent identification and development in Para athletics using open ended questions. Round One survey responses were analysed and collated into statements which formulated Round Two, where respondents were asked to rate their level of agreement with these statements. Where consensus was reached (i.e., an agreement level of >75%) no further questions were asked regarding the topic of that statement. Where consensus was not achieved, the statements were revised and carried forward to Round Three. Respondents were required to, again, rate their level of agreement with these statements.

**Results:**

A total of 23 experts completed three survey rounds (Round One, *n* = 31; Round Two, *n* = 24; Round Three *n* = 23). Thematic analysis of Round One identified themes such as quantitative assessment, coaching approaches, and physical and psychological attributes of athletes. Of 20 statements in Round Two, nine reached consensus. Of 14 statements in Round Three, seven reached consensus.

**Conclusion:**

Consensus was reached on key talent identification and development strategies including quantitative performance assessments, individualised athlete development plans and high levels of coaching experience and expertise. Together, these approaches contribute to a more structured and evidence-based pathway for athlete progression.

## Highlights

- The Paralympic movement is increasing participation rates, increasing the competitiveness of medalling opportunities and challenging resource allocation. Para sport stakeholders must strategically invest in talent identification and development to maximise their chances of sporting success.
- This is the first study to investigate the talent identification and development practices of relevant Para athletics practitioners. The three round Delphi survey highlighted key areas for consideration including quantitative assessments, athlete development, coaching approaches, trends and future directions.
- This research presents expert consensus on structuring effective talent identification and development strategies, highlighting best-practice coaching approaches that drive athlete progression and success.

## Introduction

Para sport research is a rapidly developing field (1), with a fivefold increase in Para sport research evident within the last ten years (2). Within this expanding field of research, talent identification and development (TIdD), are gaining traction. Although it may be perceived as a new practice in Para sport, TIdD is a well-established concept employed across several disciplines including the fields of business, management, education, science, technology, engineering and mathematics (STEM), and non-Para sport (3). When implemented effectively, TIdD can enhance the correct allocation of resources and increase the likelihood of return on investments (4).

Talent identification (TI) and talent development (TD) are different stages of the same process (5). The purpose of TI is to recognise those early stage athletes who have potential to progress to high performance levels (6, 7). Objective TI practices include genetic markers (8–10), performance testing (including physiological and technical skills testing), and psychological testing (11–16). Subjective TI measures include athlete comparison (against those of similar age or skill level), competition observations, and subjective measures of technical skill (17, 18). TD refers to the progression of an identified “talented’ athlete through continuous and progressive phases of a development pathway, with the ultimate goal of enabling that athlete to reach an elite level (19). Currently, there are no standard practice guidelines for TIdD in Para athletics (PA), or in any Para sport. In contrast, some non-Para sports, like soccer, have structures such as the Union of European Football Associations (UEFA) Talent Development Scheme (20) and the Fédération Internationale de Football Association (FIFA) Talent Development Ecosystem (21). These structures provide guidance for relevant stakeholders on each step of the TIdD process from increasing access to the sport to the transition of athletes into professional soccer.

In non-Para sport, youth athlete development pathways are well studied (22, 23) and conceptualised through frameworks such as the long-term athlete development model (24), and the Foundations, Talent, Elite, Mastery (FTEM) framework (25). Despite these established frameworks, early success in track and field is not necessarily an indicator for senior level success (26) which may be due to the high turnover of athletes in talent pathways (27). When considering the development of Para athletes in comparison to non-Para athletes, factors to consider include impairment onset and severity, classification (28), and the biopsychosocial factors associated with physical impairments (e.g., resilience, sport-specific skills, other life commitments) (29). Within Para sport, classification functions as a foundational structural mechanism that organises eligibility and competition while shaping athlete pathways and distributive outcomes across the Paralympic system. Classification defines who is permitted to compete and how athletes are grouped into sport classes to minimise the impact of impairment on performance (30, 31).

The age at which an athlete may engage in Para sport, and the nature of their impairment, can also influence an athlete's development (32, 33). Additionally, Para sport is associated with smaller resource pools than non-Para sport (32), and as classification is a structural determinant of Para sport, classification directly shapes resource allocation, and may be influenced by factors such as medal opportunity (32). Together, these considerations challenge TD practices in Para sport.

### Objective

The objective of this study was to examine current practices in TIdD within PA, as identified by leading experts in the field. PA refers to track-and-field sports that are adapted for athletes with physical, visual, or intellectual impairments, which is governed internationally by World Para Athletics, a division of the International Paralympic Committee. This study sought to (1) seek expert opinions regarding the effectiveness of current TIdD approaches, and (2) identify current real-world PA TIdD practices to allow coaches and sports practitioners working in PA to adopt evidence-based methods.

## Methods

### Definitions

A key requirement of a Delphi study is the recruitment of expert participants (34). For the purpose of this study, and to ensure a high level of competence among the Delphi panel, an expert was defined as an individual who has demonstrated their knowledge and experience of TIdD in PA through one or more of the following: years of experience working in PA (minimum of five years), the quantity and calibre of the athletes they have worked with, and/or formal credentials. Athletes currently competing were excluded from participating; however, previous athletes who had moved into a formal, appropriate TIdD- related role in PA were eligible.

### Panel selection

Panellists were recruited through purposeful (35) and snowball sampling (36). A call to participate in this research was administered through relevant networks, including via World Para Athletics. All those who responded to the call and who met the eligibility criteria were invited to participate. Panellists were required to have current or previous involvement in PA in a role that entails TIdD. Panellists were also required to consent to participate in the survey. The panel represented a range of ethnicities and equity-deserving groups including people living with disabilities. All panellists had equal voting rights.

### Patient and public involvement

Members of the public, who work in PA participated in this study as expert Delphi panellists. They contributed to generating and prioritising items, rating their importance, and providing feedback on consensus statements. Their input directly informed the questions in following rounds, which informed the development of the final recommendations. The research team shared the findings of this research with the panel of experts and encouraged them to aid with dissemination through their own professional networks.

### Equity, diversity and inclusion statement

This study aimed to gain global insight into current TIdD practices in PA. To achieve this, the research team prioritised recruiting panellists from diverse ethnic and geographical backgrounds. Panellists were not reimbursed for their involvement. Recruitment was conducted through publicly available professional profiles from national governing bodies and individual platforms such as LinkedIn. Additionally, Paralympics Ireland and its network of Para coaches and relevant stakeholders were engaged to promote the study nationally and internationally.

Panellists were contacted via email. Personalised reminders and flexible deadlines were used to maximise inclusion and accessibility. All members of the research team were white, non-disabled females. It is recognised that this may influence perspectives and acknowledge the potential for unconscious adoption of Western-centric views of health and independence and may underestimate the social, environmental, and emotional barriers faced by people living with impairments.

### Study design: delphi method

A Delphi study offers a structured approach to systematically gather input from a panel of experts (34, 37). Further, opting for a Delphi survey study design allowed the research team to control feedback (38), an aspect of data collection that could have been ambiguous due to the newness of TIdD in PA, and diversity within the panel.

In this study, a panel of experts on TIdD in PA independently answered three rounds of web-based surveys (39) over a seven month period (Round One was distributed in December 2024). The use of Qualtrics (Qualtrics, 2024) allowed for respondents to be based in any location ensuring data was gathered from experts across the globe. The survey was not limited by language however, panellists who agreed to participate could all speak English. There were no accessibility requirements for any panellists. Ethical approval for this study was obtained from the Human Research Ethics Committee of University College Dublin (LS-24-68). This study was registered via Open Science Framework on 9th December 2025 (https://osf.io/czu6t).

The questions included in the survey were informed by a scoping review on TIdD in PA (40). The findings of this review were summarised and presented to the panellists via a participant information leaflet prior to their consenting to participate in the study. The research team piloted the Delphi survey with three individuals familiar with the subject matter to assess clarity, face validity and logical flow of items prior to circulation to the full expert panel. Items were refined based on pilot feedback to reduce ambiguity that could introduce construct-irrelevant variance. The Delphi protocol consisted of three rounds, which is deemed to be sufficient for converging ideas and reaching consensus while maintaining participation rates (41). Consensus and stability of responses across rounds served as evidence that the iterative process had captured genuine, settled expert opinion rather than noise or random variation. Round-to-round changes in ratings were monitored, and the process concluded when responses stabilised. The research team did not have any voting rights in the consensus process. For the purpose of survey dissemination and follow up, the principal investigator (AC) was aware of the identity of the panellists. The identity of the panellists was anonymised for all other research team members (KP, AOR).

### Consensus process: round One

Round One consisted of capturing participant demographics and broad open-ended questions regarding TIdD in Para athletics. The open-ended questions enabled participants to voice their opinions on TIdD and share practices they currently employ. The questions covered topics such as the identification of a talented athlete, athlete development plans, comparative practices in non-Para athletics, trends and future directions. The results were then qualitatively assessed using thematic analysis, following the framework established by Braun and Clarke (42).

### Consensus process: round two

Round Two consisted of 20 statements generated from the thematic analysis of Round One responses, allowing participants to rank their level of agreement. The research team agreed on a pre-defined criteria for consensus, i.e., an agreement level of >75% prior to data collection. This prevents *post-hoc* rationalisation and ensures that conclusions are driven by the data rather than researcher interpretation. Further, this level of agreement has been deemed appropriate in previous research (43). To reach this level of consensus, >75% of panellists were required to rate a statement ≥4 on a 5-point Likert scale. An optional comment box was presented with each statement to allow participants the ability to provide further context to their responses.

The statements from Round Two were quantitatively assessed using percentage agreement against the agreed level of consensus (>75%). For statements that reached consensus in Round Two, no further clarifications were sought in Round Three.

### Consensus process: round three

Round Three was used to clarify statements that had not reached consensus in Round Two by presenting participants with revised statements and repeating the rating process. The same level of consensus (i.e., 75%) and quantitative assessment of percentage agreement was used. As in Round Two, participants were able to provide comments on their responses via an optional comment box linked to each statement.

## Results

### Participants

During the initial recruitment phase 57 experts who met the inclusion criteria, across 13 countries were invited to be part of the expert panel. Of these, 31 agreed to take part, resulting in the representation of eight countries. All 31 participants completed Round One of the survey, 24 participants completed Round Two and 23 participants completed Round Three. Of the 31 survey respondents, 18 (58%) were female. The majority of participants (87%) had worked with Paralympic athletes (see Table 1). Further, over half of respondents (55%) have been working in Para sport for over 16 years.

**Table 1:** Participant characteristics.

Total (*n* = 31)	
Female	18
Male	13

Role in Para athletics (*n* = 31)	
Coach	16
High performance manager	6
Classifier	3
Physiotherapist	2
Talent scout	1
Researcher/Academic	1
Skill acquisition specialist	1
Strength and conditioning coach	1

Highest level of competition (*n* = 31)	
Paralympic	27
International	2
National	1
Not applicable	1

Years of Experience (*n* = 30)	
16+ years	17
11–15 years	4
5–10 years	8
<5 years	1
Not answered	1

Paralympic Nation (*n* = 31)	
Australia	9
United Kingdom	8
Ireland	5
Canada	3
Chile	2
United States of America	2
New Zealand	1
Spain	1

### Round one

Qualitative analysis of the Round One responses (*n* = 31) identified several themes (see Supplementary File 1), that were organised into four categories: (1) quantitative assessment of talent and developmental practices, (2) athlete attributes, (3) coaching approaches and key considerations, (4) trends and future directions.

### Round two

Of the 20 statements in Round Two, nine (45%) reached consensus. The level of agreement ranged from 79.2% to 100% (see Supplementary File 2). The three statements that reached 100% agreement were related to the use of performance assessments for TI and TD, the importance of the knowledge and expertise of coaches, and the use of individualised athlete plans for TD.

When participants ranked factors to consider for TI (i.e., impairment related factors, training history, and physical characteristics), half of the respondents (50%) rated impairment related factors as the most important consideration, with training history ranked the least important (79.2% agreement). Impairment-related factors include the level of competitiveness an athlete has within their class, whether an impairment is congenital or acquired, and sport class profiles (i.e., the volume of athletes per class and the distribution of performances). Of eight psychological attributes (commitment, determination, resilience, coachability, motivation, discipline, perseverance and confidence), experts identified commitment as the most important attribute for an athlete to possess when considering sporting success, however, consensus was not reached (level of agreement = 41.2%). Over half (66.7%) of the respondents rated confidence as the least important of the eight psychological attributes for athletes to possess.

### Round three

Of 14 statements in Round Three, seven (50%) reached consensus (see Supplementary File 3). The statements which reached the highest levels of agreement were statements related to barriers to effective TIdD in PA, classification factors (i.e., how competitive an athlete is within their class), and the differences in development pathways in Para sport when compared to non-Para sport.

### Dissenting viewpoints

Seven statements related to TIdD trends in PA did not reach consensus, such as delayed event specialisation and multi-sport sampling. Only one statement, which related to training history as a factor to consider for TIdD, had an agreement of less than 50% (39.1%). The remaining six reached agreement of greater than 50%.

## Discussion

A consequence of the increased popularity of athletes in Para sport is greater competition between nations. Findings from this Delphi survey suggest the panel of experts believe effective TIdD in PA is characterised by quantitative baseline assessments, athlete specific talent development plans, and in-depth coach knowledge and experience. The strong level of agreement (>95%) regarding the need to improve TIdD practices suggests that PA stakeholders recognise and understand the importance of ensuring effective TIdD, a challenge previously reported in the literature (44).

### Quantitative assessments of talent and development practices

There was unanimous agreement amongst the experts in this study for the use of physical performance tests for the purpose of TIdD. Our survey identified that the expert panel believes objective measures of TI in PA should include performance assessments of speed, power, and endurance. This finding concurs with previous TI research in non-Para sport where it was reported that 12 out of 20 papers reviewed investigated the physical profiles of athletes via performance metrics (45). It was also the opinion of the expert panel that practitioners' priority when implementing testing measures should be the selection of tests that are specific to the event of the athlete. Practitioners can then use the test results as determinants of competitiveness and as baseline measures. A notable barrier to the effective use of performance testing in Para sport is the scarcity of normative reference data for Para athletes. In the absence of such data, practitioners may resort to comparing Para athlete data against non-Para norms, an approach that may lack appropriateness and precision. Where normative data does exist, practitioners should remain cognisant of additional factors that may compromise its accuracy, including the number of athletes within a given sports class and the range of functional capacities represented within it (46). In non-Para talent pathways, where normative data are lacking, efforts have been made to establish such normative reference data (47). In Para sport, similar efforts have been made for concussion (48) and classification norms (49). It would be prudent for Para sport research teams to undertake comparable initiatives, thereby equipping practitioners with the benchmarks necessary to evaluate Para athlete testing performances meaningfully. Further, the establishment of an international data repository could be a valuable asset for countries worldwide.

For TD, the expert panel agreed the same quantitative assessments can be used as a tool to track an athlete's progression over time. Due to the barriers of normative data detailed above, it may be more useful for practitioners to use performance tests to determine progression over time, thus comparing an athlete's data to their own historical data, not relying on potentially inappropriate comparisons.

### Athlete attributes

Our findings suggest experts believe two important psychological attributes for athletes to possess are commitment and coachability. Both attributes have previously been associated with the “coach's eye” (18), with commitment to sport found to be a favourable trait for Para athletes to possess (29). Although coachability is primarily a subjective measure, methods for quantifying athlete commitment levels have been developed and validated in non-Para sports (50–54). Other psychological attributes can be quantified via questionnaires such as the mental toughness questionnaire (55) or the psychological performance inventory (56), both of which have been validated in Para athletes (57, 58).

Based on the culmination of our research and that of others, we recommend TIdD practices should aim to quantify the psychological attributes of athletes via the Sports Commitment Model (50) and aforementioned questionnaires. Simpler practices such as attendance tracking to recognise levels of commitment, could also be implemented.

### Coaching approaches and key considerations

Our experts agreed that athlete development plans should be specific to the athlete and their needs. The panel suggests that coaches and sports practitioners need to take a holistic view of the athlete: understand the athlete's impairment, be aware of the nuances of the athlete's event, and align training with the athlete's sporting goals and aspirations. The experts further agreed that coaches should work with their athletes to set specific goals and perform subsequent goal assessments. Suggested development plan formats to follow included established models such as the Long-Term athlete development plan (24), or the Bondarchuk model (59). Alternatively, TD plans should be time specific, i.e., annual or daily performance plans.

It was identified in this research that the knowledge and expertise of coaches is the most crucial factor for TD. It was agreed that the lack of coaches with PA knowledge is a primary barrier to effective TIdD. Previous research on TD in Para sport has reported that coaches have a high level of influence on a Para athletes' training processes (60); that a key element of successful TD is coach qualification level (61, 62); and that coaches should hold a combination of sport-specific and impairment-specific knowledge (63). Despite this, Para sport coaches face gaps in formal education opportunities (64). Further, a recent mixed methods investigation reported that PA athletes tend to opt for coaching support that reflects their needs: athletes with low care needs typically seek coaches with international sporting credentials, whereas athletes with high care or equipment needs, such as wheelchair racers or runners with guides, seek coaches who understand their impairment over sporting expertise (65). It should be noted that the impairment-specific knowledge in question does not relate to the pathophysiology of an athlete's impairment but to how the impairment presents for the athlete and how it may impact their ability to perform sporting tasks. There is an onus on Para sport practitioners to share their knowledge and expertise not only with coaches in their own national Para sport team, but across research groups and with athletes of similar impairments (66). The aforementioned suggestion of an international data repository could be a means of enabling this knowledge share.

Developmental differences between Para and non-Para athletes are well established (32, 33) with further variation evident within Para sport according to whether an impairment is acquired or congenital (28, 67). Para athletes must also navigate classification and potential reclassification processes, the latter of which can jeopardise medal prospects and consequently influence resource allocation (68). Despite these developmental disparities and the complexities associated with classification, the expert panel agreed that their underlying coaching principles do not differ meaningfully between athletes with and without impairments. However, this similarity primarily referred to the philosophical or pedagogical orientation of coaching**,** rather than suggesting that training environments or operational practices were identical. These coaching principles included approaches to training progression, athlete–coach communication, and performance planning. In practice, coaching within PA often requires substantial adaptation related to impairment-specific demands, equipment use, and support structures.

Given the recognised importance of sport for individuals living with impairments (69, 70), and the current scarcity of Para sport coaches, this finding supports the notion that the fundamental principles underpinning coaching need not differ based on the presence of a physical impairment. Rather, the panel supports the coaching approach of seeking to align the physical and psychological capacities of the athlete with the specific demands of their event, with consideration given to the implementation of training sessions, technical instruction, and performance support.

### Trends and future directions

Multi-sport sampling enables young athletes to cultivate a broad range of movement skills while mitigating the risk of injury and psychological burnout (71–73). This approach may hold particular value for Para athletes whose opportunities to develop movement capabilities during childhood may have been compromised by exclusion from school-based physical education (74). Furthermore, multi-sport sampling has been associated with delayed event specialisation, a practice increasingly recognised as more advantageous for adolescent athletes (7, 75). Both multi-sport sampling and delayed event specialisation share conceptual overlap with talent transfer (76, 77), which has similarly emerged as a notable trend within Para sport. Despite the weight of these findings, the expert panel failed to reach consensus on statements pertaining to the growing prevalence of multi-sport sampling or the relative merits of delayed vs. early specialisation. This may reflect the time typically required for research evidence to translate into applied practice, particularly within school and grassroots club settings. The experts of this study agreed that the future of TIdD in PA will be characterised by advancements in technology and artificial intelligence (AI), with future methods including more sophisticated biomechanical analyses and improved equipment capabilities. Indeed, the use of AI for TI in Paralympic sport has already been implemented for future Games cycles: Australia launched their YouFor2032 campaign in 2025 (April, 2025) (78), which included the roll out of a TI mobile application which employs AI to determine the eligibility of the user for various Para sporting events.

In comparison to such AI tools, improved equipment capabilities may take longer to be implemented into practice. Previous research has found that, although Para athletes find assistive equipment helpful, it can oftentimes be a barrier to participation (79), with some sports classes at distinct disadvantages due to the level of equipment needs associated with their event (80). Therefore, collaboration between sporting bodies, rehabilitation and disability services is essential.

### Research implications

The findings of this survey add to the growing body of research in Para sport. Stakeholders in PA should apply these findings into practice to improve the effectiveness of their TIdD protocols. To optimise the practical application of these findings, stakeholders should be invited to engage with research teams, acting as a form of Patient and Public Involvement (PPI). PPI encourages collaboration between researchers and practitioners or participants, ensuring that research outcomes are relevant, practical, and aligned with real-world needs. Through this process, stakeholders can help determine which practices are most feasible to implement and which are best suited for their specific contexts.

### Limitations

While the Delphi method facilitated consensus on some statements, it also highlighted a number of limitations. Firstly, it inherently restricted the number of questions that could be included and required prioritisation of topics, which may have limited the breadth of discussion. Secondly, the use of an online survey format did not allow for in-person discussion that might have enabled deeper exploration and clarification. Thirdly, selection bias may have influenced the findings, as most respondents were coaches, potentially excluding valuable perspectives from other TIdD experts. Additionally, the impact of non-responders on the representativeness of the sample is unknown, which may have introduced biases. Future work could include non-Para experts to determine whether transferable insights exist across athlete populations.

Participant attrition across rounds constitutes a limitation of the present study. It is plausible that some individuals chose to withdraw following disagreement with particular statements, meaning that responses gathered in later rounds may disproportionately reflect the views of those more closely aligned with the emerging consensus. This selective attrition may have introduced response bias, with the potential to have influenced the overall findings.

The expert panel is subject to certain limitations, including a disproportionate representation of coaches, a high concentration of Paralympic-level expertise, and a potential Western bias. Consequently, the generalisability of our findings may be restricted to populations similar to those represented within this panel.

A further limitation of this research is its focus exclusively on TIdD experts, which precluded the inclusion of current athletes. This decision reflects the premise that athletes represent the “talent” being identified and developed, rather than those responsible for these processes. It is worth noting, however, that a number of the experts consulted had prior experience as athletes themselves. Future research would benefit from exploring athlete perspectives on their lived experiences of TIdD in sport, with a particular focus on identifying which practices are most prevalent and perceived as most beneficial.

## Conclusion

Successful TIdD is multifactorial and should account for quantitative assessments and athlete development while also considering coach characteristics. It was agreed that quantitative performance assessments such as speed, power, and endurance testing, specific to the athletic event, should be used for TIdD. Experts believe the results of these quantitative tests should be monitored across time and compared to elite performance standards. It was suggested that athlete development plans, regardless of the model used, should be athlete specific. It was agreed that the knowledge and expertise of PA coaches is a crucial factor in TIdD, and a major barrier to effective TIdD is the limited availability of such coaches. Lastly, the expert panel agreed that multi-sport sampling and advancements in technology are key trends that Para sport practitioners should be aware of.

## Data Availability

The original contributions presented in the study are included in the article/Supplementary Material, further inquiries can be directed to the corresponding author.
