# Supplementary material for: From guesswork to guidance: a delphi study on practices of talent identification and development in Para athletics
Source: Front Sports Act Living. 2026 May 11;8:1791259. doi: 10.3389/fspor.2026.1791259 (PMC13200201; doi:10.3389/fspor.2026.1791259)
Supplement: Supplementary file 1 [file Supplementaryfile1.docx]

| **Themes** | **Panel responses** |
| --- | --- |
| Quantitative assessment of talent and developmental practices | “Identifying key variables by combining sport knowledge with expertise in classification”  “Performance testing: speed test, power test, endurance tests, time trials, baseline”  “Training program (integration of the principle of specificity and overload (allowing for progression over time)), mapping of progression against set goals, adaptability, evidence based/ using programs with established success/ scientific backing”  “Exposure to elite environments, training camps and appropriate competition” |
| Athlete attributes | “Athlete characteristics include commitment to high performance and independence, attitude, growth mindset/grit, motivation”  “Training age/ training history (including training pre impairment if acquired, training with AB groups), athlete age, talent transfer”  “Willingness to commit to being an elite athlete, determination, resilience, listens to coach, motivation, discipline, perseverance, passion for their sport, confidence in their event(s)”  “Good external support - Supportive family and club/facilities, no differentiation between siblings with/without impairments” |
| Coaching approaches and key considerations | “Relative competitiveness within their sports class, suitability for their event”  “Correct resource allocation: right coach, right facility, right teammates, right equipment”  “Geographical considerations, access to opportunities, closeness to facilities and competition”  “Knowledge of the coaches: years of experience, third level education, disability specific knowledge, ability to be adaptable”  “Coached according to the physical and psychological demands of their event, it does not change from Para to non-Para” |
| Trends and future directions | “Sport sampling, suitability for a sport and late specialisation”  “Lack of research and data available on athletes due to the range of impairments, lack of longitudinal data, female and junior data”  “Advancements in technology and AI - determining classification and equipment capabilities, looking at neurodiversity, report generation, biomechanical analysis”  “Increased focus on psychological measures including psycho-social considerations of being a para athlete” |

Figure 1.

Round One Themes
