# Supplementary figures and images for: From guesswork to guidance: a delphi study on practices of talent identification and development in Para athletics
Source: Front Sports Act Living. 2026 May 11;8:1791259. doi: 10.3389/fspor.2026.1791259 (PMC13200201; doi:10.3389/fspor.2026.1791259)

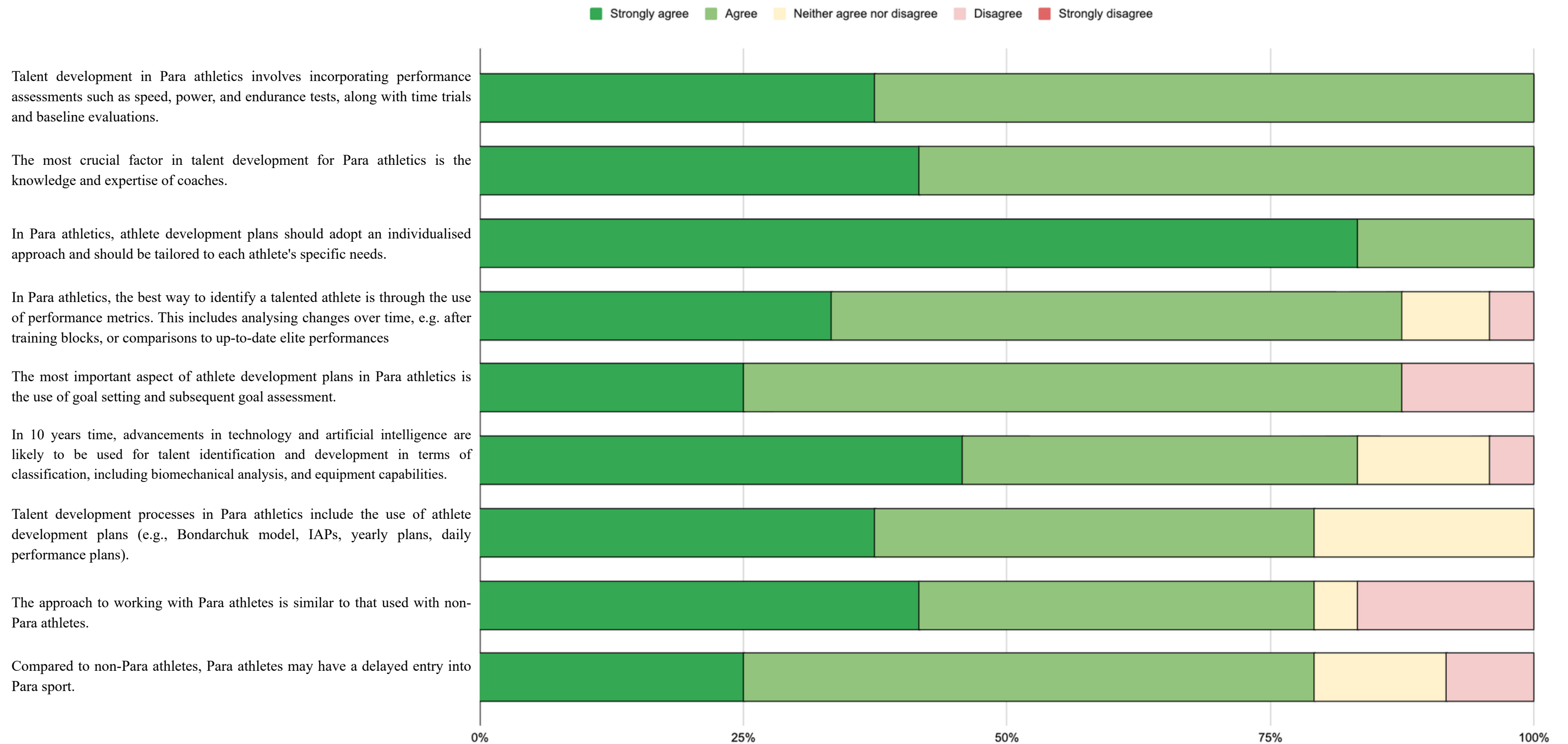

Figure 2.  
Round Two consensus statements.

Supplement: Supplementary file 2 [file Supplementaryfile2.pdf]

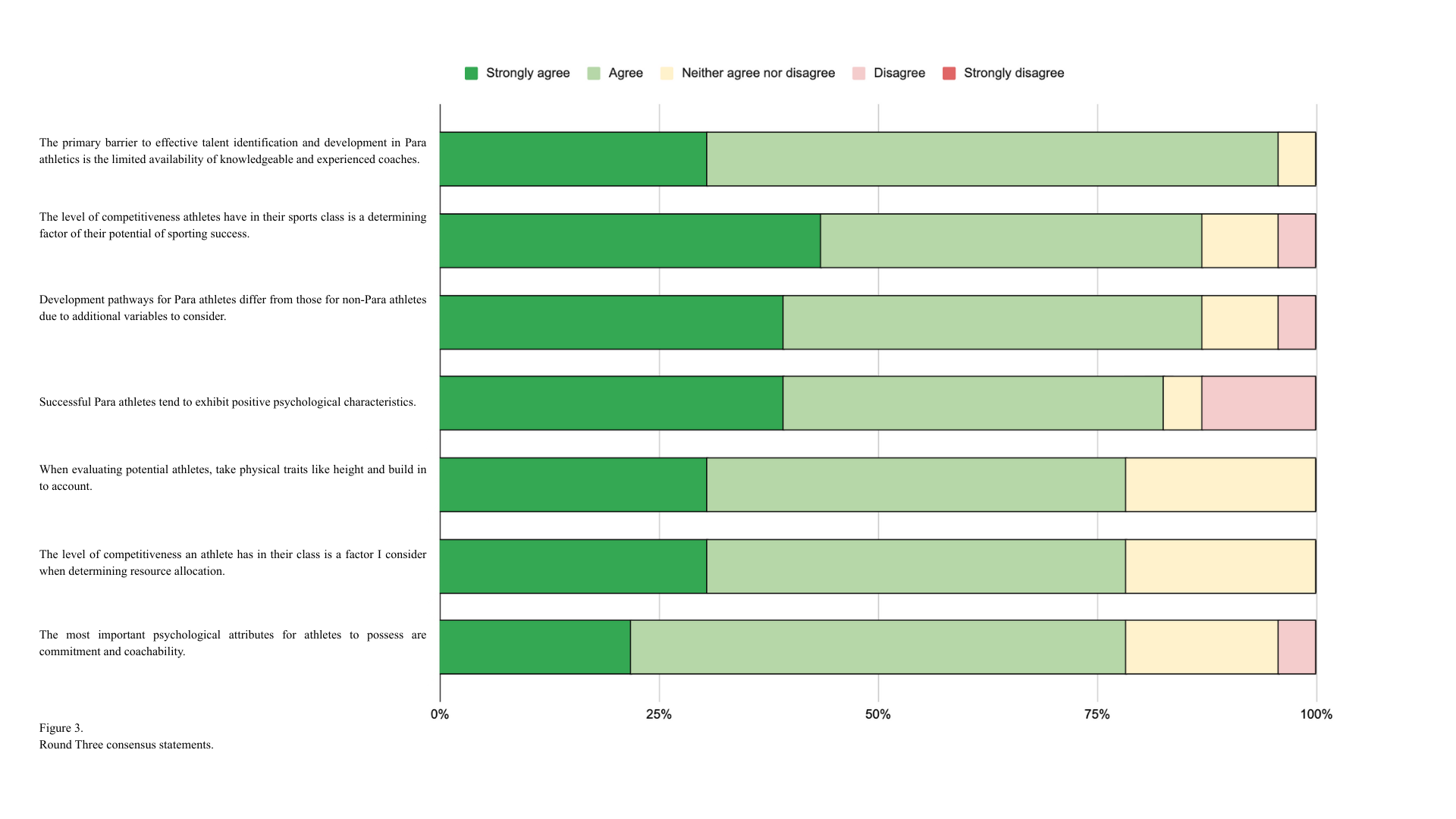

Supplement: Supplementary file 3 [file Supplementaryfile3.png]
